# Community Vs. hospital HIV testing sites in Jerusalem, Israel - who’s tested and who’s at risk?

**DOI:** 10.1186/s13584-020-00368-3

**Published:** 2020-05-18

**Authors:** Dor Atias, Hagai Levine, Hila Elinav, Michele Haouzi-Bashan, Yotam Lior, Zohar Mor

**Affiliations:** 1 The Hebrew University Hadassah Medical School, Jerusalem, Israel; 2 The Jerusalem Open House for Pride and Tolerance, Jerusalem, Israel; 3 Braun School of Public Health, Hebrew University-Hadassah, Jerusalem, Israel; 4 Hadassah AIDS center, department of clinical microbiology and infectious diseases, Hadassah Hebrew University medical center, Jerusalem, Israel; 5 The Soroka Clinical Research Center, Soroka University Medical Center, Beer-Sheva, Israel; 6 Tel Aviv Department of Health, Tel Aviv, Israel; 7 School of Health Sciences, Ashkelon Academic College, Ashkelon, Israel

**Keywords:** AIDS, Gays, Men who have sex with men, Migrants, Primary prevention, Sexual behavior

## Abstract

**Background:**

After decades of constant increase in HIV diagnoses among men who have sex with men (MSM), a gradual decrease has been reported in recent years. Timely detection of HIV leads to early treatment and behavioral changes which decrease further transmissions. This cross-sectional study aimed to assess demographic and behavioral characteristics of individuals who were tested for HIV in Jerusalem, Israel.

**Methods:**

This study compared individuals who were tested at Hadassah AIDS Center (HAC) with those tested at the Jerusalem Open House (JOH) - an LGBTQ community center. Participants completed anonymous questionnaires regarding their demographic, HIV-testing history, and sexual behaviors. High-risk sexual behavior (HRSB) was defined as a diagnosis of sexually transmitted disease or condomless anal/vaginal sex during the last year.

**Results:**

Among 863 participants, 104 (12.1%) were tested in HAC and 759 (87.9%) in JOH. Of those, 19 (18.3%) and 227 (29.9%) were HRSB, respectively. Two MSM were tested positive in JOH. JOH received more MSM, HRSB and individuals who were previously tested for HIV, while HAC received more migrants and health-care workers. HRSB-participants were more commonly younger, males, non-Jewish, with lower income, previously tested for HIV, reported more sexual partners, payed for sex or used drugs.

**Conclusions:**

MSM and HRSB-individuals were more likely to be tested in JOH, while migrants and health-care workers in HAC, possibly due to the geographic location, reputation and specific atmosphere. In order to encourage HIV-tests among HRSB and non-Jews, additional interventions should be employed, including outreach activities, extending opening hours and reducing testing costs should be employed.

## Background

After more than two decades of constant increase in HIV diagnoses among men who have sex with men (MSM), a gradual decrease has been reported in recent years both in Europe and Israel [1–3]. Yet, the burden of disease among MSM remains high and recent reports estimated that the proportion of MSM among all men infected with HIV in Israel to be as high as 41% [4]. Several behavioral characteristics are associated with the high proportion of HIV among MSM, including AIDS optimism, the use of recreational drugs during sex- which is colloquially termed as Chemsex, and the increasing use of geographical network applications to seek sexual partners [5]. As a response to the burden of HIV, MSM and other key risk groups for HIV infection are encouraged to use condoms and recommended to test periodically for HIV. Additional innovative strategies include the use of pre-exposure prophylaxis (PrEP) in selected cases and encouraging people who are living with HIV/AIDS to increase their adherence to the antiretroviral therapy (ART) [6].

Early HIV detection through periodic HIV testing enables infected individuals to use ART at early stages of their infection and therefore to suppress their viral load and decrease the risk for further HIV transmission [7, 8]. The Joint United Nations Program on HIV/AIDS (UNAIDS) set a goal in December 2013 to reach that 90% of people who are HIV infected will be diagnosed, 90% of people who are diagnosed will be on ART and 90% of those who receive ART will be virally suppressed, which is conceptualized as the 90–90-90 cascade [9].

Confidential HIV tests in Israel are performed free of charge by primary care physicians, community organizations or the AIDS centers in general hospitals. Individuals who are interested in anonymous testing can attend one of the seven AIDS centers distributed throughout Israel or any of the few testing sites operated by a non-governmental organization (NGO) in the community, located in Tel-Aviv, Jerusalem and Haifa. NGO community testing sites are often characterized by a friendly, non-stigmatizing atmosphere and are mostly operated by peers from the key risk groups themselves. An Israeli study performed 20 years ago showed that individuals from key risk groups prefer to be tested at community testing sites rather than in a hospital setting [10].

The Jerusalem district comprised of over 1 million inhabitants, 66.5% of them are Jewish, 30.7% Muslims, 1.5% Christians and 1.3% are undefined [11]. Two HIV testing sites are certified and regulated by the Israeli ministry of health (MOH) in Jerusalem. The first is the Hadassah AIDS center at Hadassah hospital (HAC), which is located outside of the city center and operates once a week by nurses and doctors during the morning hours. The second is the Open clinic at the Jerusalem Open House for Pride and Tolerance (JOH), an NGO based testing site located in the city center. It is operated by peers from the Lesbian, Gay, Bisexual, Transgender, and Queer (LGBTQ) community and offers HIV testing once weekly in the evening hours. While both centers offered free HIV testing, they differ in their protocol of operation. At HAC, only anonymous testing require payment, while at JOH all testing required payment until July 2018 and were offered free of charge since then.

As these two testing centers in Jerusalem are different in their operating procedures and ambiance, this study aimed to compare the demographic characteristics, sexual behaviors and testing results of individuals who were tested in JOH and HAC and characterize the high-risk sexual behavior among individuals who are tested for HIV in Jerusalem. Results from this study can be used by NGOs and the MOH to target populations at risk and appropriate testing infrastructure for the needs and preferences of the targeted populations.

## Methods

This cross-sectional study was performed in two testing sites in Jerusalem, the JOH and HAC, between October 2017 and November 2018. All individuals older than 14 years of age who visited the testing these sites were requested to complete the anonymous study questionnaire. Participants were asked to indicate their demographic details regarding their sex, age, religion, city of residence, income, level of education, as well as their sexual preferences and behaviors, substances use, history of HIV testing and the reason for the current test. Independent variables for this study included high-risk sexual behavior (HRSB) and the choice for a specific HIV testing site - either JOH or HAC. The definition of HRSB was aligned with the indications set by the Centers for Diseases Control and Prevention for PrEP use [12], which included prior diagnoses of sexually transmitted disease (STD) in the last year and/or whether they were MSM and performed condomless sex in the last year, or were heterosexuals and performed anal or vaginal condomless sex in the last year with a person at risk for HIV transmission. Person at risk for HIV transmission included MSM, immigrants from Africa and intravenous drug users.

### Statistical analysis

Participants who were tested in JOH were compared with those who were tested in HAC, and participants who were involved in HRSB in the last year were compared with those at low risk. Continuous variables were compared by the Student’s *t*-test if normally distributed, while the Mann Whitey non-parametric test was performed in other cases. Categorical variables were compared by the chi-square test or Fisher exact test when the number of variables in the cells was less than five. Variables that reached statistical significance lesser than 5% in the univariate analysis were included in the multivariate analyses performed by logistic regression model after exclusion of variable interactions to associate demographic and behavioral characteristics of individuals who prefer to get tested at the JOH.

## Results

During the study period, 244 individuals were tested in HAC and 775 in the JOH. Of those, 104 (42.6%) and 759 (97.9%) completed the study questionnaire, respectively. The average age of all 863 participants who completed the questionnaire was 30.4 ± 10.8 years and the majority (81.8%) were males. Two individuals (0.3%) were tested positive for HIV in JOH, both were MSM, living in Jerusalem, underwent previous HIV tests and reported infrequent condom use during sex. They did not report prior STD diagnosis, nor did they pay for sex or preformed sex under the influence of drugs during the past year.

Individuals who were tested at the JOH had a higher level of education and were more likely to be previously tested for HIV than individuals who were tested in HAC (Table 1). JOH had higher proportion of MSM and likely to engage in HRSB, HAC had higher proportion of migrants from Africa. Individuals who were tested in JOH indicated that the reason for choosing the testing site was the atmosphere at the JOH (although results did not reach statistical significance), while those tested at HAC more commonly indicated the credibility of the site, which is situated in the hospital.

**Table 1:** Demographic and behavioral characteristics of individuals tested in the JOH^a^Vs. the HAC^b^

Variable (number of responders)		JOH^a^*n* = 759 (87.9%)	HAC^b^*n* = 104 (12.1%)	*P*
Mean age (±standard deviation)		30.3 ± 10.8	31.3 ± 10.5	0.4
Male sex (*n* = 863)		622 (81.9)	78 (75.0)	0.09
None Jew (*n* = 647)		96 (17.7)	14 (13.5)	0.3
Living outside of Jerusalem (*n* = 863)		54 (7.1)	9 (8.7)	0.6
Above high-school education (*n* = 590)		360 (72.7)	58 (61.1)	0.02
Income higher than the average salary (*n* = 552)		94 (20.6)	15 (15.8)	0.3
Prior HIV test (*n* = 852)		531 (70.9)	56 (54.4)	0.001
Risk groups (*n* = 863)	MSM^c^	390 (51.4)	22 (21.2)	< 0.001
	Immigrants from African countries	4 (0.5)	9 (8.7)	< 0.001
	Sex workers	2 (0.3)	0 (0.0)	1.0
	Intravenous drug users	3 (0.4)	0 (0.0)	1.000
	Partners of people from risk groups	32 (4.2)	4 (3.8)	1.0
	Not belonging to any key risk group	328 (43.2)	69 (66.3)	< 0.001
High risk (*n* = 863)		227 (29.9)	19 (18.3)	0.01
Reason for testing (*n* = 863)	Condomless sex	378 (49.8)	48 (46.2)	0.5
	New relationship	104 (13.7)	15 (14.4)	0.8
	Routine test	203 (26.7)	20 (19.2)	0.1
	Occupational exposure	2 (0.3)	6 (5.8)	< 0.001
	Intra venous drug use	0 (0.0)	1 (1.0)	0.1
	Paid for sex	11 (1.4)	0 (0.0)	0.4
	Other low-risk reasons for testing	15 (2.0)	6 (5.8)	0.03
Reasons for choosing the testing center (*n* = 862)	Easy access	369 (48.6)	54 (52.4)	0.5
	Rapid tests available	216 (28.5)	27 (26.2)	0.6
	Credibility	147 (19.4)	39 (37.9)	< 0.001
	Atmosphere	143 (18.8)	12 (11.7)	0.07
	Recommendations from friends	133 (17.5)	13 (12.6)	0.2
	Cost	52 (6.9)	6 (5.8)	0.7
	Not associated with any population	65 (8.6)	8 (7.8)	0.8
Positive HIV results (*n* = 759)		2 (0.3)	0 (0.0)	1.000

^a^JOH – Jerusalem Open House for Pride and tolerance

^b^HAC – Hadassah AIDS center

^c^MSM – Men who have Sex with Men

In the multivariate analysis (Table 2), higher levels of education and being MSM were associated with choosing JOH as a testing site for HIV, while being a migrant from Africa or being at a lower sexual risk for HIV transmission were negatively associated with JOH. In our study, 246 (28.5%) participants were involved in HRSB during the last year. They were found to be more commonly younger, males, non-Jewish, living outside of Jerusalem, had lower income and were previously tested for HIV compared with those who reported low sexual risk for HIV transmission (Table 3). Individuals involved in HRSB also reported a greater number of sexual partners, were more likely to pay for sex, performed sex under the influence of recreational drugs and were tested because they performed condomless sex compared with individuals who were at a low risk for HIV transmission.

**Table 2:** Variables predicting testing for HIV at JOH^a^

Variable	Odds ratio	95% Confidence Interval	*P*
Above high-school education	1.7	1.1–2.7	0.04
Prior HIV test	1.2	0.7–1.9	0.4
Risk Group – MSM^b^	3.1	1.7–5.5	< 0.001
Risk Group - Immigrants from African countries	0.1	0.02–0.5	0.006
Reason for testing – other low-risk reason	0.3	0.1–1.1	0.07
High-risk sexual behavior	1.0	0.5–1.9	0.9

^a^*JOH* Jerusalem Open House for Pride and tolerance

^b^*MSM* Men who have Sex with Men

**Table 3:** Demographic and behavioral characteristics of high-risk Vs. low-risk participants

Variable (number of responders)		High risk *n* = 246 (28.5%)	Low risk *n* = 617 (71.5%)	*P*
Mean age (±standard deviation)		28.4 ± 11.2	31.2 ± 10.6	0.001
Male sex (*n* = 863)		228 (92.7)	472 (76.5)	< 0.001
None Jew (*n* = 647)		47 (25.7)	63 (13.6)	< 0.001
Living outside of Jerusalem (*n* = 863)		26 (10.6)	37 (6.0)	0.02
Above high-school education (*n* = 590)		116 (67.1)	302 (72.4)	0.2
Income higher than the average salary (*n* = 552)		21 (13.3)	88 (22.3)	0.02
Prior HIV test (*n* = 852)		186 (76.5)	401 (65.8)	0.002
Sexual behavior in the last year	More than 15 sexual partners (*n* = 715)	22 (11.2)	23 (4.4)	0.001
	Paid for sex (*n* = 793)	19 (8.1)	25 (4.5)	0.04
	Having sex under the influence of psychoactive drugs (*n* = 803)	89 (36.8)	157 (28.0)	0.01
Reason for testing (*n* = 863)	Condomless sex	167 (67.9)	259 (42.0)	< 0.001
	New relationship	21 (8.5)	98 (15.9)	0.005
	Routine test	48 (19.5)	175 (28.4)	0.007
	Occupational exposure	0 (0.0)	8 (1.3)	0.1
	Intra venous drug use	0 (0.0)	1 (0.2)	1.0
	Paid for sex	3 (1.2)	8 (1.3)	1.0
	Other low-risk reasons for testing	2 (0.8)	19 (3.1)	0.05
Reasons for choosing the testing center (*n* = 862)	Easy access	125 (50.8)	298 (48.4)	0.5
	Rapid tests available	57 (23.2)	186 (30.2)	0.04
	Credibility	55 (22.4)	131 (21.3)	0.7
	Atmosphere	42 (17.1)	113 (18.3)	0.7
	Recommendations from friends	44 (17.9)	102 (16.6)	0.6
	Cost	17 (6.9)	41 (6.7)	0.9
	Not associated with any population	19 (7.7)	54 (8.8)	0.6
Positive HIV results (*n* = 759)		2 (0.8)	0 (0.0)	0.08

## Discussion

This study indicated that individuals who were tested at the JOH were more commonly MSM and involved in HRSB, while two (0.3%) were detected with HIV, both were MSM. Those who were tested in HAC were more commonly migrants and their sexual behavior was at lower risk compared with those tested at the JOH.

MSM and individuals who reported HRSB were more likely to be tested in community-based testing center rather than testing sites which are situated in hospital, in agreement with findings from other studies from Israel and other countries [10, 13, 14]. These preferences can be attributed to two main factors. First, the JOH functions as an LGBTQ community center, which provides additional services for MSM, such as public events, psychological treatments, and social support. It operates by LGBTQ peers and provides a friendly and non-stigmatized space for sexual minorities. The non-stigmatizing atmosphere is effective in improving the access to health care among the LGBTQ community who may face stigmatization or discomfort while interacting with health care workers [15, 16]. Second, the JOH is geographically situated in the city center, while the HAC do not offer HIV testing after working hours and is located in a suburb of the city which is relatively difficult to access.

The HAC testing site captured different populations than JOH, such as health care workers who were tested following occupational exposures in the hospital and migrants from African. Participants in our study rated the HAC as having higher credibility than the JOH, which is attributed to the nature of a hospital setting. The HAC employs Ethiopian health worker who supports the migrants and provides linguistic translation and cultural adaptation, in addition to translated printed materials in Amharic which are available at this site. Other publications have also demonstrated the association between the capability of a testing center to respond to the cultural and linguistic barriers of the migrants which are associated with patient-physician relationship, increase the frequency of testing and supporting prevention interventions [17–19].

The HIV prevalence among the HRSB population in our study was 0.8%, which correlates with the current estimations of HIV prevalence among MSM in Israel [20]. However, compared with previous studies performed in an STD clinic in Tel-Aviv, we have found a lower proportion of HRSB among the study participants, such as previous STD diagnoses, purchasing sex and condomless sex [21–23]. It might be that MSM at high risk in Jerusalem were tested in other places, like Tel Aviv, or that the JOH did not capture them. The cost of HIV tests which was required at the JOH until July 2018 might also discouraged HRSB individuals with lower income to be tested in JOH.

The proportion of non-Jewish individuals in our study was lower than their actual proportion in the Jerusalem district (17.0 and 33.5%, respectively) [11], although relatively higher proportion of non-Jews were involved in HRSB. A recent study performed in Israel found that Arab MSM demonstrated inferior knowledge regarding HIV transmission compared with Jewish MSM. The same study also demonstrated that Arabs were more likely than Jews to engage in HRSB [24]. This underrepresentation of Arabs who were tested for HIV should be addressed by both HIV testing centers in Jerusalem while taking into consideration that non-Jewish MSM in Jerusalem may be more traditional and ‘closeted’ in comparison with the Jewish population, and therefore more difficult to reach.

As shown in our study, socio-cultural adaptations of testing sites increase the centers accessibility to target populations. Therefore, we recommend the Jerusalem testing sites to continue developing culturally competent interventions to enhance HIV testing among MSM and individuals who practice HRSB, as well as among non-Jewish individuals and immigrants from African countries. This can be achieved by reducing possible clinic-related barriers for testing for example, expanding working hours, offering free HIV tests and providing anonymous and rapid testing [25, 26]. Advertising the centers services could be useful in attracting hard to reach population. This can be achieved by outreach activities in gay-related venues and immigrant centers or by promoting the clinic’s services via geosocial networking smartphone applications which are frequently used by MSM to seek their sexual encounters [27, 28]. Lastly, as a response to the increasing rates of STDs and the prevalence of HIV-STDs co-infection [29–31], we also recommend expanding the HIV testing centers to provide a complete STD panel, anonymously and affordably.

This is the first study to assess HIV-testing sites in Jerusalem, yet, it is subject to several limitations. First, information bias is possible, as participants may be uncomfortable to share their sexual experiences. To reduce this bias, the questionnaires were completed anonymously. Second, due to the different intake protocols between JOH and HAC, response rates between the centers were different. At JOH, completion of the questionnaire was part of the routine intake at the clinic, while in the HAC the questionnaires were offered to the individuals in addition to other registration procedures, therefore the compliance in HAC was lower and might have resulted in selection bias. To assess the possible bias, we compared the age and sex of the HAC testees who participated in the study with the total population that was tested in HAC in the same period. Individuals who participated in the study were younger (31.2 versus 35.1 years, *p* = 0.01) and more commonly male (75.0% versus 65.0%, *p* = 0.07). This selection bias is therefore conservative, as older individuals and females are probably engaging in lower sexual risk behavior [32]. Third, the difference in HIV testing costs and procedural change practiced at JOH in July 2018 limits the investigators to assess the association between the cost and the adherence to HIV testing. To assess the effect of the policy change on the composition of JOH population, we compared those who were tested prior and after the pricing policy change. No significant difference in age, sex, religion and risk score were found (Additional file 1). Forth, PrEP has been approved for use in Israel during the study period and requires HIV testing before administration at their family physicians. This policy change could have potentially shifted HRSB individuals from NGO testing sites to the community clinics, a move that may have resulted in underrepresentation of the HRSB population, and therefore the selection bias, if exists, is conservative.

## Conclusions

Higher proportion of MSM and those who performed HRSB were tested at the JOH, while migrants were more likely to be tested at HAC. Social and cultural adaptations of the testing site are effective in capturing population at high risk for HIV transmission. In order to improve HIV prevention programs in Jerusalem, both centers should facilitate out-reach interventions aiming HRSB individuals and the non-Jewish population.

## Supplementary information

**Additional file 1 Table 1** Demographic and behavioral characteristics of individuals tested in the JOH* before the pricing policy change Vs. after the pricing policy change.

## Data Availability

Can be applied upon request from the correspondent author.

## Abbreviations

AIDS: Acquired immunodeficiency syndrome
ART: Antiretroviral therapy
HAC: Hadassah AIDS Center in Hadassah hospital
HIV: Human immunodeficiency virus
HRSB: High-risk sexual behavior
JOH: Jerusalem Open House for Pride and Tolerance
LGBTQ: Lesbian, Gay, Bisexual, Transgender, and Queer
MOH: Israeli ministry of health
MSM: Men who have sex with men
NGO: Non-governmental organization
PrEP: Pre-exposure prophylaxis
STD: Sexually transmitted disease
UNAIDS: The Joint United Nations Program on HIV/AIDS
