# Supplementary material for: Community Vs. hospital HIV testing sites in Jerusalem, Israel - who’s tested and who’s at risk?
Source: Isr J Health Policy Res. 2020 May 18;9:10. doi: 10.1186/s13584-020-00368-3 (PMC7232836; doi:10.1186/s13584-020-00368-3)
Supplement: Supplementary file 1 — Additional file 1 Table 1 Demographic and behavioral characteristics of individuals tested in the JOH* before the pricing policy change Vs. after the pricing policy change. [file 13584_2020_368_MOESM1_ESM.docx]

Table 1 – Demographic and behavioral characteristics of individuals tested in the JOH* before the pricing policy change *Vs*. after the pricing policy change

| Variable (number of responders) | | JOH* before the change  n=561 (73.9%) | JOH* after the change n=198 (26.1%) | *P* |
| --- | --- | --- | --- | --- |
| Mean age (±standard deviation) | | 30.7 ± 11.0 | 29.1 ± 10.2 | 0.5 |
| Male sex (n=759) | | 462 (82.4) | 160 (80.8) | 0.6 |
| None Jew (n=543) | | 70 (17.6) | 26 (17.8) | 0.9 |
| Living outside of Jerusalem (n=759) | | 41 (7.3) | 13 (6.6) | 0.7 |
| Above high-school education (n=495) | | 270 (74.8) | 90 (67.2) | 0.09 |
| Income higher than the average salary (n=457) | | 74 (22.1) | 20 (16.4) | 0.2 |
| Prior HIV test (n=749) | | 383 (69.1) | 148 (75.9) | 0.07 |
| Risk groups (n=759) | MSM^$^ | 274 (48.8) | 116 (58.6) | 0.02 |
|  | Immigrants from African countries | 2 (0.4) | 2 (1.0) | 0.3 |
|  | Sex workers | 2 (0.4) | 0 (0.0) | 0.4 |
|  | Intravenous drug users | 0 (0.0) | 3 (1.5) | 0.02 |
|  | Partners of people from risk groups | 25 (4.5) | 7 (3.5) | 0.6 |
|  | Not belonging to any key risk group | 258 (46.0) | 70 (35.4) | 0.009 |
| High risk (n=759) | | 168 (29.9) | 59 (29.8) | 0.9 |
| Reason for testing (n=759) | Condomless sex | 286 (51.0) | 92 (46.5) | 0.3 |
|  | New relationship | 85 (15.2) | 19 (9.6) | 0.051 |
|  | Routine test | 135 (24.1) | 68 (34.3) | 0.005 |
|  | Occupational exposure | 2 (0.4) | 0 (0.0) | 1.000 |
|  | Intra venous drug use | 0 (0.0) | 0 (0.0) | NA |
|  | Paid for sex | 9 (1.6) | 2 (1.0) | 0.7 |
|  | Other low-risk reasons for testing | 11 (2.0) | 4 (2.0) | 0.9 |
| Sexual behavior in the last year | More than 15 sexual partners (n=629) | 29 (6.1) | 12 (7.6) | 0.5 |
|  | Paid for sex (n=699) | 33 (6.4) | 9 (5.0) | 0.5 |
|  | Having sex under the influence of psychoactive drugs (n=709) | 166 (31.4) | 49 (27.1) | 0.3 |
| Reasons for choosing the testing center (n=759) | Easy access | 276 (49.2) | 93 (47.0) | 0.6 |
|  | Rapid tests available | 167 (29.8) | 49 (24.7) | 0.2 |
|  | Credibility | 103 (18.4) | 44 (22.2) | 0.2 |
|  | Atmosphere | 100 (17.8) | 43 (21.7) | 0.2 |
|  | Recommendations from friends | 94 (16.8) | 39 (19.7) | 0.3 |
|  | Cost | 39 (7.0) | 13 (6.6) | 0.9 |
|  | Not associated with any population | 47 (8.4) | 18 (9.1) | 0.8 |
| Positive HIV results (n=759) | | 2 (0.4) | 0 (0.0) | 1.000 |

*JOH – Jerusalem Open House for Pride and tolerance

^$^MSM – Men who have Sex with Men
